# Investigation and Management of Lower Limb Septic Arthritis in Children: An Update Using the Latest British Orthopaedic Association Standard for Trauma (BOAST) Guidelines

**DOI:** 10.7759/cureus.62179

**Published:** 2024-06-11

**Authors:** Alexandra Sebastiao, Haseeb Khawar, Russell Hawkins

**Affiliations:** 1 Trauma and Orthopedics, Whittington Health NHS Trust, London, GBR; 2 Trauma and Orthopedics, Nottingham University Hospitals NHS Trust, Nottingham, GBR; 3 Orthopedic Surgery, Royal Cornwall Hospitals NHS Trust, Truro, GBR

**Keywords:** pediatric orthopedics, knee and hip, lower limb, joint infection, sepsis, septic arthritis, child

## Abstract

Septic arthritis is a serious condition in children, with the hip and knee joints most typically affected. Patients typically present with pain, joint swelling, fever, and an inability to bear weight. Early recognition and treatment are crucial, as untreated septic arthritis can lead to serious complications, including sepsis, irreversible joint damage, growth problems, and early-onset arthritis. Clinical signs, inflammatory markers, and imaging are used for the diagnosis of septic arthritis. The mainstay of management includes antibiotic therapy and surgical washout. Long-term follow-up is essential to monitor for complications.

## Introduction and background

Septic arthritis is an infection that develops in a synovial joint, with hematogenous spread of bacteria being the most common etiology in children [1]. It is a serious diagnosis in children presenting with a painful joint and has significant morbidity and mortality. Documented mortality was as high as 50% prior to the use of antibiotics in the management of septic arthritis, compared to more recent studies showing mortality rates of less than 1% in children who receive treatment [2]. Complications can range from systemic features such as sepsis to specific bony pathology including osteomyelitis and irreversible damage to joint cartilage [3]. Growth disturbance due to damage to the growth plate may also occur in children [4]. Manz et al. [5] describe long-term effects affecting growth and bone deformity in 2-40% of children with septic arthritis. The importance of early detection and prompt treatment is highlighted by the recently published British Orthopaedic Association Standard for Trauma (BOAST) on the management of pediatric musculoskeletal infections [6]. The primary aim of our literature review is to present the up-to-date epidemiology, clinical features, classification, investigations, and management options for children with septic arthritis of the lower limb based on the recent BOAST standard, to ensure hospital doctors are aware of this potentially debilitating condition. The secondary aim of our review is to consolidate our findings into a flowchart to act as an aide memoire for the hospital doctor to ensure children with septic arthritis are treated as per the BOAST standard.

## Review

Epidemiology

The incidence of septic arthritis in children is 2-7 per 100,000 per year in the Western world [7-8]. The highest incidence is seen in children under four years old [9] and it affects males more than females with a ratio of about 1.7 [1]. Other risk factors include prematurity, low birth weight, immunosuppression, and respiratory distress syndrome [2]. Septic arthritis most commonly affects the large joints in the lower limbs, with hips and knees being the most common [1]. *Staphylococcus aureus* is the most common causative organism [2] accounting for 40-60% of cases [10-12]. Other common organisms include group *A Streptococcus* and *Enterobacter* [2]. Children with sickle cell disease are at higher risk of infections with *Salmonella* [13]. *Kingella Kingae* is a pathogen more frequently being found in children under 36 months [14-15].

Clinical features

The classical presentation of a child with septic arthritis of the lower limb includes an acutely swollen, painful, warm hip or knee joint associated with fever, systemic upset, and restriction of movement [16]. The child may also be unable to bear weight or walk with a limp. Children may present with local symptoms only or local and systemic symptoms. It is important to note that a subacute picture is very common with more subtle and non-specific signs and symptoms [17].

Examination 

Any child suspected of having septic arthritis should receive a thorough examination. Observations should be taken as soon as possible, with a raised temperature, elevated heart rate, low blood pressure, or low consciousness being concerning features. As well as a full examination of the painful joint in question, the joints above and below should also be examined. Hip pain is commonly referred to as the knee in children, and hip pathology will easily be missed if only the knee is examined [18]. As well as this, any child with suspected septic arthritis should also have an examination of their spine and a full systemic assessment to exclude alternative infection sources [6]. This should include upper respiratory tract and ears [19]. Generally, the limb will be held in a position where the joint capsule is most lax for comfort. For a hip, this will be in a position of flexion, abduction, and external rotation, whereas an affected knee will be held in a slightly flexed position [3]. There may be warmth and sometimes erythema overlying the joint. An effusion may be palpable in subcutaneous joints such as the knee or ankle. The child will usually hold the joint in their fixed position of comfort, with a reluctance to actively move it, and severe pain exhibited on any attempt at passive movement.

Investigations

**Figure 1:**
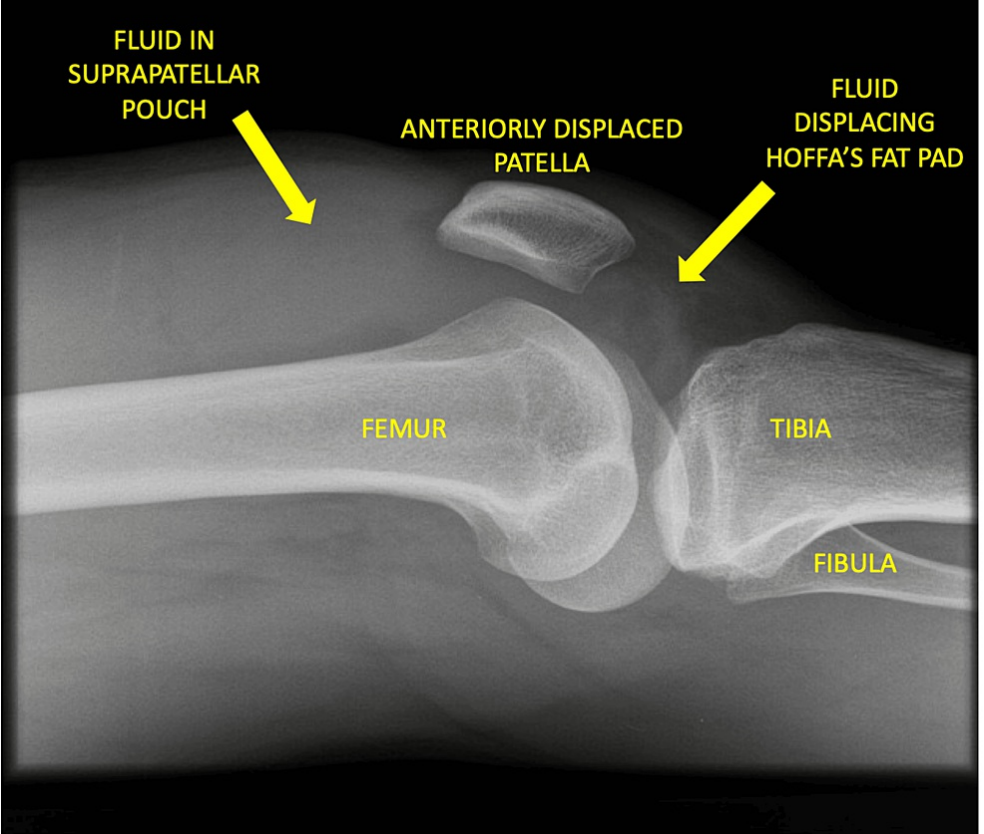
Plain lateral X-ray of the knee showing a joint effusion Credit: Image adapted from Radiopaedia [23]. Published under Creative Common License.

**Figure 2:**
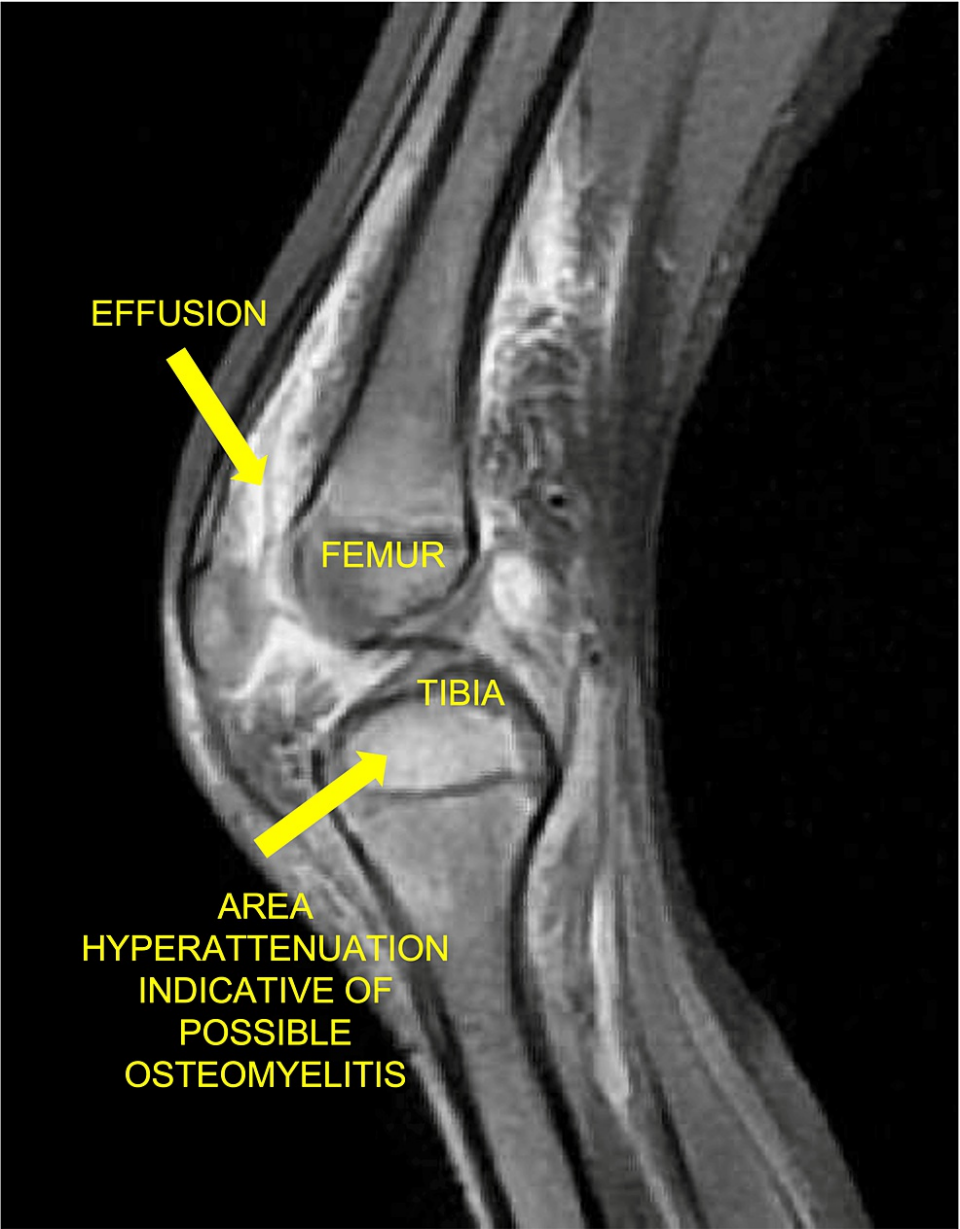
MRI knee of a child with septic arthritis Credit: Image adapted from Radiopaedia [24]. Published under Creative Common License.

**Figure 3:**
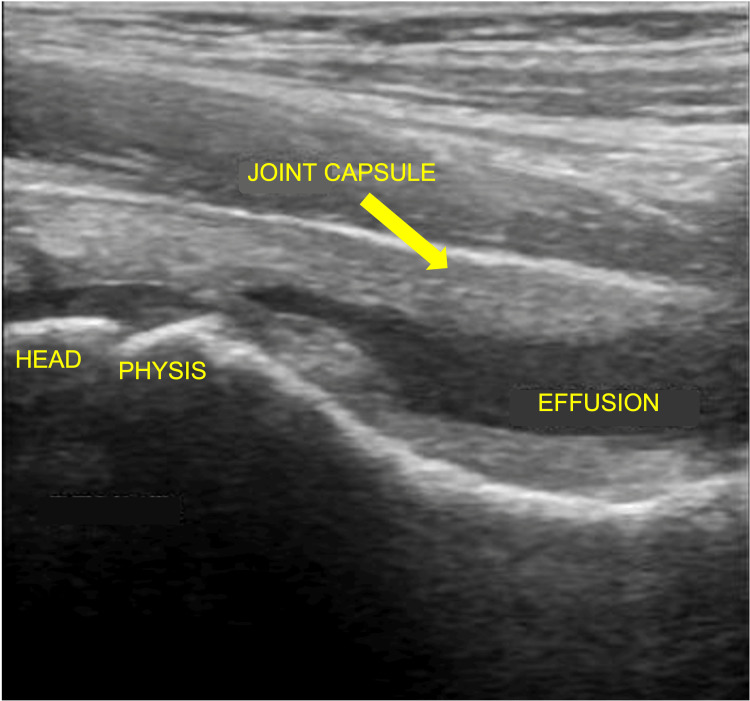
Ultrasound showing joint effusion of the hip Credit: Image adapted from Radiopaedia [25]. Published under Creative Common Licence.

A child presenting with signs or symptoms of septic arthritis should be admitted to the hospital and receive joint inpatient care from both orthopedics and pediatric specialists [6]. Key investigations include a full blood count (FBC), C-reactive protein (CRP), erythrocyte sedimentation rate (ESR), and blood cultures. Imaging must include orthogonal plain radiographs of the affected joint and adjacent bones to exclude other pathology. An X-ray demonstrating a knee effusion is shown in Figure 1. MRI may be conducted as second-line imaging if the patient is stable, or there is diagnostic doubt, and should be done within 48 hours of presentation [6]. MRI is the most reliable imaging modality to determine any associated osteomyelitis and bone changes [20]. Figure 2 shows an MRI of a patient with septic arthritis of the knee. Ultrasound may be an alternative when MRI is not possible [19] and may be more sensitive than MRI at detecting early joint effusions [20]. An ultrasound image showing an effusion of the hip joint is shown in Figure 3. If a child has acute symptoms and initial investigations reveal elevated inflammatory markers or joint effusion on imaging, they should be kept nil by mouth and urgently referred to orthopedics. It is important to note that normal inflammatory markers do not exclude septic arthritis early in the disease course [19]. If the child is clinically stable and not displaying signs of hemodynamic compromise from sepsis, antibiotic therapy may be delayed allowing joint aspiration first to obtain a sample for microscopy and culture [21]. Fluid should be sent in both universal culture pots and blood culture bottles [19]. Synovial fluid sampling must be done by a trained clinician and may require ultrasound guidance. A causative organism is found in around one-third of children with septic arthritis, and this information may be utilized later to determine the type and duration of antibiotic therapy [22]. However, if the child is unwell or displaying any parameters of septic shock, then antibiotic therapy must not be delayed [19].

Classification

Kocher’s criteria is a tool that can be used to determine the likelihood of septic arthritis over transient synovitis in a child presenting with a painful hip [26-27]. The modified Kocher’s criteria include CRP as an additional significant predictor of septic arthritis (Table 1) [28].

**Table 1:** Kocher’s criteria, modified by Caird et al. Reference: [26-28] CRP: C-reactive protein; ESR: erythrocyte sedimentation rate

Modified Kocher criteria (1 point each)	Likelihood of septic arthritis
Fever >38.5°C	1 point - 37%
Cannot weight-bear	2 points - 62%
ESR >40mm	3 points - 83%
WBC >12 x10^9/L	4 points - 93%
CRP >20	5 points - 98%

Differential diagnosis

Transient synovitis is the most common cause of a painful hip in children [29] and is a self-limiting condition [30]. It is often challenging to differentiate between transient synovitis and septic arthritis as they can present similarly [28]. The most reliable clinical signs for distinguishing septic arthritis from transient synovitis are a temperature of >38.5°C and an inability to weight-bear [27-26]. Other differentials to consider in a child presenting with a limp would be a slipped capital femoral epiphysis (SCFE) and Perthes disease [3]. These can largely be excluded with imaging. Inflammatory arthropathies such as reactive arthritis and juvenile idiopathic arthritis can be difficult to distinguish at the first presentation of monoarthritis. Poor response to antibiotic therapy, presence of symptoms for more than six weeks, and polyarticular involvement indicate that an inflammatory cause is more likely [31]. It is always important to consider trauma and non-accidental injury, particularly in pediatric patients presenting with a limp [32]. Pyomyositis, particularly surrounding the hip joint, can have a similar presentation to septic arthritis, and MRI would be the imaging modality to distinguish these presentations [19].

Management

Empirical antibiotics should be commenced immediately in any child who meets the NICE criteria for high-risk sepsis [6,33]. These high-risk criteria are age-dependent and include factors such as behavioral changes, heart rate, and respiratory rate. The criteria are summarized in Table 2. If the child is stable and surgery is planned, antibiotics may be delayed until synovial fluid sampling is completed [6]. Osteomyelitis in adjacent bones must be considered in all cases of septic arthritis [19].

**Table 2:** NICE criteria for high-risk sepsis, summarized by age group Reference: [6,33] NICE: National Institute for Health and Care Excellence

	Age <5	Age 5-11	Age 12-17
Behavior	No response to social cues OR Appears ill to a healthcare professional OR Does not wake, or if roused does not stay awake OR Weak high-pitched or continuous cry	Objective evidence of Altered behavior or mental state OR Appears ill to a healthcare professional OR Does not wake, or if roused does not stay awake	Objective evidence of new altered mental state
Heart rate (in beats per minute)	Age <1 year: ≥160 OR Age 1–2 years: ≥150 OR Age 3–4 years: ≥140 OR <60 at any age	Age 5 years: ≥130 OR Age 6–7 years: ≥120 OR Age 8–11 years: ≥115 OR <60 at any age	All ages: >130
Respiratory rate (in breaths per minute)	Age <1 year: ≥60 OR Age 1–2 years: ≥50 OR Age 3–4 years: ≥40 OR Grunting OR Apnea OR Oxygen saturation of <90% in air or increased oxygen requirement over baseline	Age 5 years: ≥29 OR Age 6–7 years: ≥27 OR Age 8–11 years: ≥25 OR Oxygen saturation of <90% in air or increased oxygen requirement over baseline	All ages: ≥25 OR New need for ≥40% oxygen to maintain saturation >92% (or >88% in known chronic obstructive pulmonary disease)
Additional features	Mottled or ashen appearance OR Cyanosis of skin, lips, or tongue OR Non-blanching rash of skin OR Temperature: <3 months: ≥38°C OR Any age: <36°C	Mottled or ashen appearance OR Cyanosis of skin, lips, or tongue OR Non-blanching rash of skin	Not passed urine in previous 18 hours, or for catheterized patients passed <0.5 ml/kg of urine per hour OR Mottled or ashen appearance OR Cyanosis of skin, lips or tongue OR Non-blanching rash of skin
Systolic blood pressure (in mmHg)	N/A	N/A	All ages: 90≥ OR >40 below normal

**Figure 4:**
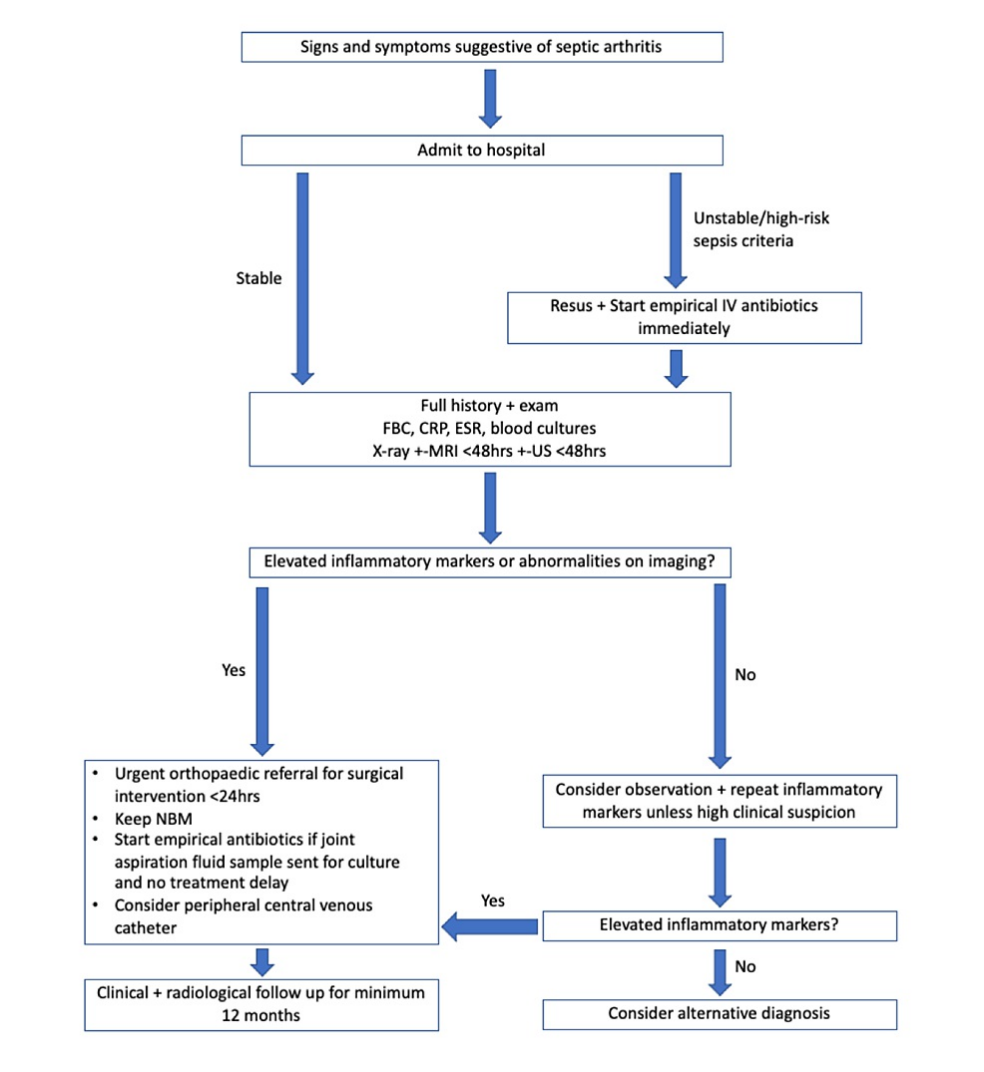
Suggested treatment algorithm for managing septic arthritis FBC: full blood count; CRP: C-reactive protein; ESR: erythrocyte sedimentation rate; NBM: nil by mouth

Definitive management comprises urgent surgical drainage and lavage of the joint [2], ideally within 24 hours. Once a synovial fluid sample has been taken, high-dose empirical antibiotics may be commenced. Antibiotics can be tailored if culture and sensitivity results become available. A peripherally inserted central venous catheter should be considered early if conversion to oral therapy is not appropriate so IV antibiotics can be administered on an outpatient basis [6]. IV antibiotics can be converted to oral antibiotics when inflammatory markers fall and the child improves clinically [19]. Antibiotics should be given at least until inflammatory markers are back to normal; however, the overall length of antibiotic treatment should be determined on a local multidisciplinary team (MDT) basis [19]. Patients must receive clinical and radiological follow-up from a clinician for at least 12 months to identify any long-term complications or growth disturbances after any episode of septic arthritis [6]. The authors suggested algorithm for managing septic arthritis is shown in Figure 4.

## Conclusions

Septic arthritis in children is an orthopedic emergency that can have significant long-term complications if not treated promptly. The hip and knee joints are most commonly affected in the lower limb. Diagnosis should be considered in any child presenting with a painful joint or non-weight bearing. Key clinical features include inability to bear weight, pain, fever, and restriction of movement. Initial investigations include blood for inflammatory markers along with imaging to identify any joint effusion and exclude other pathologies. Differentiating between septic arthritis and transient synovitis of the lower limb joints can be challenging. Management must be led by orthopedic or pediatric specialists and involves surgical drainage and lavage of the joint with antibiotic therapy. A follow-up is required to monitor for long-term complications.

## References

[1] Krogstad P (2004). Osteomyelitis and septic arthritis. Textbook of Pediatric Infectious Diseases.

[2] Kang SN, Sanghera T, Mangwani J, Paterson JM, Ramachandran M (2009). The management of septic arthritis in children: systematic review of the English language literature. J Bone Joint Surg Br.

[3] Wall C, Donnan L (2015). Septic arthritis in children. Aust Fam Physician.

[4] Ilharreborde B (2015). Sequelae of pediatric osteoarticular infection. Orthop Traumatol Surg Res.

[5] Manz N, Krieg AH, Buettcher M, Ritz N, Heininger U (2020). Long-term outcomes of acute osteoarticular infections in children. Front Pediatr.

[6] (2023). BOAST - the management of children with acute musculoskeletal infection. https://www.boa.ac.uk/resources/boast-the-management-of-children-with-acute-musculoskeletal-infection.html.

[7] Faust SN, Clark J, Pallett A, Clarke NM (2012). Managing bone and joint infection in children. Arch Dis Child.

[8] Peltola H, Vahvanen V (1984). A comparative study of osteomyelitis and purulent arthritis with special reference to aetiology and recovery. Infection.

[9] Okubo Y, Nochioka K, Marcia T (2017). Nationwide survey of pediatric septic arthritis in the United States. J Orthop.

[10] Goergens ED, McEvoy A, Watson M, Barrett IR (2005). Acute osteomyelitis and septic arthritis in children. J Paediatr Child Health.

[11] Wang CL, Wang SM, Yang YJ, Tsai CH, Liu CC (2003). Septic arthritis in children: relationship of causative pathogens, complications, and outcome. J Microbiol Immunol Infect.

[12] Yuan HC, Wu KG, Chen CJ, Tang RB, Hwang BT (2006). Characteristics and outcome of septic arthritis in children. J Microbiol Immunol Infect.

[13] al-Salem AH, Ahmed HA, Qaisaruddin S, al-Jam'a A, Elbashier AM, al-Dabbous I (1992). Osteomyelitis and septic arthritis in sickle cell disease in the eastern province of Saudi Arabia. Int Orthop.

[14] Moumile K, Merckx J, Glorion C, Pouliquen JC, Berche P, Ferroni A (1992). Bacterial aetiology of acute osteoarticular infections in children. Acta Paediatr Oslo Nor.

[15] Lundy DW, Kehl DK (1998). Increasing prevalence of Kingella kingae in osteoarticular infections in young children. J Pediatr Orthop.

[16] Pääkkönen M (2017). Septic arthritis in children: diagnosis and treatment. Pediatric Health Med Ther.

[17] Gill P, Sanders JE (2019). Emergency department management of pediatric septic arthritis and osteomyelitis. Pediatr Emerg Med Pract.

[18] Houghton KM (2009). Review for the generalist: evaluation of pediatric hip pain. Pediatr Rheumatol Online J.

[19] Mitchell PD, Abraham A, Carpenter C (2023). Consensus guidelines on the management of musculoskeletal infection affecting children in the UK. Bone Joint J.

[20] Manz N, Krieg AH, Heininger U, Ritz N (2018). Evaluation of the current use of imaging modalities and pathogen detection in children with acute osteomyelitis and septic arthritis. Eur J Pediatr.

[21] Pääkkönen M, Kallio MJ, Peltola H, Kallio PE (2010). Pediatric septic hip with or without arthrotomy: retrospective analysis of 62 consecutive nonneonatal culture-positive cases. J Pediatr Orthop B.

[22] Lyon RM, Evanich JD (1999). Culture-negative septic arthritis in children. J Pediatr Orthop.

[23] Dixon A (2024). Joint effusion | radiology reference article | radiopaedia.org. https://radiopaedia.org/cases/2696.

[24] (2024). Septic arthritis of the knee - paediatric | radiology case | radiopaedia.org. https://radiopaedia.org/cases/89918.

[25] Patel MS (2024). Hip joint effusion (ultrasound) | radiology case | radiopaedia.org. https://radiopaedia.org/cases/41679.

[26] Kocher MS, Zurakowski D, Kasser JR (1999). Differentiating between septic arthritis and transient synovitis of the hip in children: an evidence-based clinical prediction algorithm. J Bone Joint Surg Am.

[27] Kocher MS, Mandiga R, Zurakowski D, Barnewolt C, Kasser JR (2004). Validation of a clinical prediction rule for the differentiation between septic arthritis and transient synovitis of the hip in children. J Bone Joint Surg Am.

[28] Caird MS, Flynn JM, Leung YL, Millman JE, D'Italia JG, Dormans JP (2006). Factors distinguishing septic arthritis from transient synovitis of the hip in children. A prospective study. J Bone Joint Surg Am.

[29] Landin LA, Danielsson LG, Wattsgård C (1987). Transient synovitis of the hip. Its incidence, epidemiology and relation to Perthes' disease. J Bone Joint Surg Br.

[30] Asche SS, van Rijn RM, Bessems JH, Krul M, Bierma-Zeinstra SM (2013). What is the clinical course of transient synovitis in children: a systematic review of the literature. Chiropr Man Therap.

[31] Aupiais C, Basmaci R, Ilharreborde B (2017). Arthritis in children: comparison of clinical and biological characteristics of septic arthritis and juvenile idiopathic arthritis. Arch Dis Child.

[32] Wilson E, Cox P, Greaves K, Paul SP (2018). Recognition and nursing management of children with non-traumatic limp. Emerg Nurse.

[33] (2023). Recommendations | sepsis: recognition, diagnosis and early management | guidance | NICE. https://www.nice.org.uk/guidance/ng51/chapter/Recommendations.

